# Age-stratified comparative analysis of the differences of gut microbiota associated with blood glucose level

**DOI:** 10.1186/s12866-019-1466-y

**Published:** 2019-05-27

**Authors:** Wu Enqi, Zhao Huanhu, Wu Ritu, Xie Dan, Lin Han, Wang Baili, Shen Gangyi, Li Shuchun

**Affiliations:** 1 School of Pharmacy, Minzu University of China, 27 South Street, Zhongguancun, Beijing, 100081 China; 2 Key Laboratory of Ethnomedicine (Minzu University of China), Ministry of Education, Beijing, 100081 China; 3 School Hospital, Minzu University of China, Beijing, 100081 China

**Keywords:** Gut microbiota, Age, Blood glucose level, 16S rRNA-based high-throughput sequencing

## Abstract

**Background:**

Gut bacteria are an important component of the microbiota ecosystem in humans and other animals, and they play important roles in human health. The aim of this study was to investigate the relationship between gut microbiota and multiple demographical-, behavioral-, or biochemical-related factors in subjects with chronic disease. Subjects with a very wide age range who participated in community-based chronic disease prevention and screening programs in China were enrolled. We analyzed the intestinal microbiota composition using 16S rRNA-based high-throughput sequencing of fecal samples, analyzed the association between gut microbiota structure and multiple demographical, behavioral, and biochemical factors, and compared the differences in microbiota composition in age-stratified groups with different blood glucose levels.

**Results:**

Our results showed that both age and blood glucose levels had a significant impact on the gut microbiota structure. We also identified several taxa showed distinct abundance in groups with different glucose levels. *Lactobacillus* and *Bifidobacterium* at genus level and their related taxa were more abundant in the GLU high group comparing with GLU normal group and in NGR group comparing with DM group. Further analysis using the age-stratified data showed that blood glucose levels had a more significant impact on the gut microbiota in the ≥76 y age group than in the ≤75 y age group, which indicated that it is necessary to take age into account when conducting such studies. Moreover, we identified several taxa that were highly associated with blood glucose levels in the ≥76 y age group but not in the ≤75 y age group. Within the ≥76 y age group, Lachnospiraceae *incertae sedis* and *Bacteroides* were more abundant in the GLU normal group, whereas *Lactobacillus* and *Bifidobacterium* at genus level were more abundant in the GLU high group.

**Conclusions:**

This result suggested that taxa that are capable of differentiating blood glucose levels might differ significantly in different age groups.

## Background

Diabetes is a common metabolic disease characterized by hyperglycemia resulting from defects in insulin secretion, insulin action, or both. [1]. Worldwide, 415 million people live with diabetes, and an estimated 193 million people have undiagnosed diabetes. China is on pace to become the country with the highest population of diabetics in the world, with 103 million people diagnosed. Type 2 diabetes (T2D) accounts for more than 90% of patients with diabetes [2] and leads to microvascular and macrovascular complications that cause blindness, kidney failure, lower limb amputation, etc. The care and treatment of diabetics places considerable socioeconomic pressures on the medical system.

Gut bacteria are an important component of the microbiota ecosystem in humans and other animals, and they play important roles in human health, such as nutrient absorption, homeostatic control of energy balance, immunoregulation, gastrointestinal development, and many other physiological processes. Gut bacteria can mirror host physiology [3]. Gastrointestinal microbiota represent a complex ecosystem with enormous diversity. Gut microbiota not only participate in the synthesis of dietary fatty acids and the absorption of fat-soluble vitamins, but also affect the colonization of pathogenic bacteria and regulate bile acid transformation, consequently regulating energy homeostasis [4–6]. The diversity and composition of gut microbiota are also affected by host physiological factors, hereditary factors, and dietary and environmental factors [7, 8]. Dysbiosis of gut bacterial communities is associated with many chronic diseases, such as type I diabetes (TID), obesity, inflammatory bowel disease (IBD), rheumatoid arthritis, cancer, autism, and allergies [9]. Several studies have provided evidence that the pathogeneses of type 2 diabetes (T2D), such as chronic low-grade inflammation and insulin resistance, are significantly associated with intestinal microbiota compositional changes, which cause increased absorption of monosaccharides, increased production of insulin resistance-related substances, changes in intestinal lining permeability, and increased production of lipopolysaccharides (LPS) [10–12]. The differences between the composition of the intestinal microbiota in humans with T2D and non-diabetic persons [10, 13–20] indicate that T2D is associated with differences in Actinomycetaceae, *Alistipes*, *Bacteroides*, Betaproteobacteria, *Bifidobacterium*, Clostridia, Coriobacteriaceae, Desulfovibrionaceae, Erysipelotrichaceae, Eubacterium, *Faecalibacterium*, Firmicutes, *Fusobacterium*, Lachnospiraceae, *Lactobacillus*, *Parabacteroides*, Peptostreptococcaceae, Planococcaceae, *Prevotella*, Propionibacteriaceae, Proteobacteria, *Roseburia*, *Streptococcus*, Veillonellaceae, and Verrucomicrobia. These studies provide evidence of an association between intestinal dysbiosis and T2D. However, there are great discrepancies across studies with respect to taxa changes in T2D patients as compared with healthy controls. This inconsistency could be explained by different sequencing technologies, different statistical methods, and the selection of thresholds of significance. However, ineffective control of confounding factors that might affect the association between microbiota and the target population also contributes to the observed inconsistency. It is evident that the activity and composition of the gut microbiota change with advancing age [21]. Aging is considered a chronic inflammation process [22], and dysbiosis plays a pivotal role in the pathogenesis and development of age-related diseases including T2D [23]. However, to our best knowledge, studies evaluating the effects of age-related factors on the relationship between intestinal microbiota and T2D are lacking.

In this study, we investigated the relationship between gut microbiota and plasma glucose levels, age, as well as multiple demographical-, behavioral-, and biochemical-related factors in subjects with a very wide age range who participated in community-based chronic disease prevention and screening programs in China. In addition, we compared the composition of gut microbiota in groups with different plasma glucose levels after age stratification.

## Results

### Characteristics of the participants

Samples from a total of 133 participants including 55 males and 78 females were investigated in the study. The major demographic, clinical, and behavioral characteristics of the participants are shown in Table 1. The age of the participants ranged from 44 to 88. According to WHO diagnostic criteria for diabetes (1999), 78 participants who had fasting plasma glucose < 6.1 were included in the normal blood glucose group (NGR). Among the 55 participants who were included in the high blood glucose group (HGR), 22 had fasting plasma glucose level between 6.1 and 7.0 and were categorized as the impaired fasting glucose group (IFG), while 33 had fasting plasma glucose level no less than 7.0 and were categorized as the diabetic group (DM). Except for the fasting plasma glucose level, all other variables were comparable between NGR and HGR, or between NGR, IGF, and DM groups.

**Table 1:** Demographic, biochemical and behavior characteristics of the participants

		Two Categorical		Three Categorical			Overall
		GLU normal (*n* = 78)	GLU high (*n* = 55)	NGR(*n* = 78)	IFG(*n* = 22)	DM(*n* = 33)	
Numerical Variables							
Age (year)		69.08 ± 10.14	69.22 ± 10.04	69.08 ± 10.14	67.55 ± 10.80	70.33 ± 9.51	69.14 ± 10.06
BMI (kg/m^2^)		25.5 ± 3.31	26.25 ± 3.02	25.5 ± 3.31	26.35 ± 3.04	26.18 ± 3.05	25.82 ± 3.2
WHR		0.85 ± 0.07	0.88 ± 0.07	0.85 ± 0.07	0.87 ± 0.07	0.88 ± 0.07	0.87 ± 0.07
GLU (mmol/L)		5.37 ± 0.42	7.76 ± 1.66	5.37 ± 0.42	6.47 ± 0.23	8.62 ± 1.65	6.36 ± 1.62
CHO (mmol/L)		5.21 ± 1.05	5.17 ± 1.15	5.21 ± 1.05	5.04 ± 1.22	5.25 ± 1.10	5.19 ± 1.08
HDLC (mmol/L)		1.48 ± 0.38	1.36 ± 0.27	1.48 ± 0.38	1.36 ± 0.23	1.36 ± 0.30	1.43 ± 0.34
LDLC (mmol/L)		3.23 ± 0.78	3.31 ± 0.99	3.23 ± 0.78	3.17 ± 1.08	3.4 ± 0.94	3.26 ± 0.87
TG (mmol/L)		1.91 ± 1.12	1.9 ± 1.38	1.91 ± 1.12	2.03 ± 1.70	1.82 ± 1.14	1.91 ± 1.23
SBP (mmHg)		80.77 ± 10.95	78.16 ± 10.07	80.77 ± 10.95	79.23 ± 11.32	77.45 ± 9.26	79.69 ± 10.63
DBP (mmHg)		140.24 ± 20.17	135.64 ± 17.22	140.24 ± 20.17	140.55 ± 20.63	132.36 ± 13.91	138.34 ± 19.08
Categorical Variables							
Gender(*n* = 133)	male	27	28	27	10	18	55
	female	51	27	51	12	15	78
Edu3 (*n* = 123)	lower than college	25	15	25	8	7	40
	college or higher	46	37	46	13	24	83
Exercise (*n* = 99)	more than once a week	38	34	38	12	22	72
	less than once a week	16	11	16	6	5	27
Meat (*n* = 128)	not prefer	37	21	37	10	11	58
	normal or prefer	38	32	38	12	20	70
Fruit (*n* = 127)	not prefer	27	23	27	9	14	50
	normal or prefer	47	30	47	13	17	77
Vegetable (*n* = 126)	not prefer	9	4	9	1	3	13
	normal or prefer	64	49	64	21	28	113
Alcohol (*n* = 127)	more than 4 days a week	3	3	3	2	1	6
	1–3 days a week	2	3	2	2	1	5
	less than ones a week	69	47	69	18	29	116
Smoke (*n* = 121)	not exposed	49	29	49	13	16	78
	exposed 4 or more days a week	13	20	13	8	12	33
	exposed 1–3 days a week	6	4	6	1	3	10
Sleeping (*n* = 124)	less than 6 h a day	18	18	18	7	11	36
	more than 6 h a day	55	33	55	13	20	88
Sleepless (*n* = 115)	more than 3 days a week	8	5	8	1	4	13
	1–3 days a week	7	10	7	5	5	17
	less than once a week	51	34	51	13	21	85
Stress (*n* = 131)	happy	44	33	44	13	20	77
	normal	29	18	29	7	11	47
	unhappy	3	4	3	2	2	7

### Overall assessment of intestinal microbiota

A total of 25,375 K PE-reads of the 16S rDNA gene V3–V4 region were generated from the 133 specimens, with an average of 19,0791.3 (±39,560.7 SD) reads for each specimen, ranging from 85,363 to 368,127. A total of 19,305 K high quality PE-reads were obtained after trimming and filtering. In the OTU clustering process, a total of 37,668 sequences of chimeras were filtered and 7033 OTUs were yielded. After alignment of the OTU representative sequences using the QIIME pipeline, a total of 1366 OTUs were included for further data analysis.

In the taxonomical assignment process with a confidence threshold of 80%, 1366 operational taxonomic units (OTUs) were identified and annotated. Among these, 1302 OTUs at the phylum level had an annotation reliability over 0.8 and covered 16 phyla; 1231 OTUs at the class level had an annotation reliability over 0.8 and covered 28 classes; 1206 OTUs at the order level had an annotation reliability over 0.8 and covered 45 orders; 1056 OTUs at the family level had an annotation reliability over 0.8 and covered 99 families; and 652 OTUs at the genus level had an annotation reliability over 0.8 and covered 232 genera.

Firmicutes (47.9%) and Bacteroidetes (37.8) were found to be the dominant taxa at the phylum level, as were Clostridia (38.8%) and Bacteroidia (37.8%) at the class level and Clostridiales (38.8%) and Bacteroidales (37.8%) at the order level. At the family level, Bacteroidaceae (27.3%), Lachnospiraceae (21.0%), and Ruminococcaceae (15.6%) were the most dominant taxa covering more than 10% of the overall reads. At the genus level, a total of 53 genera had an abundance of more than 0.1%; among them, *Bacteroides* (27.3%), *Prevotella* (6.6%), *Faecalibacterium* (6.6%), *Roseburia* (5.2%), and *Escherichia*/*Shigella* (5.1%) were the most dominant taxa covering more than 5% of the overall reads.

### Explanatory variables analysis

**Fig. 1:**
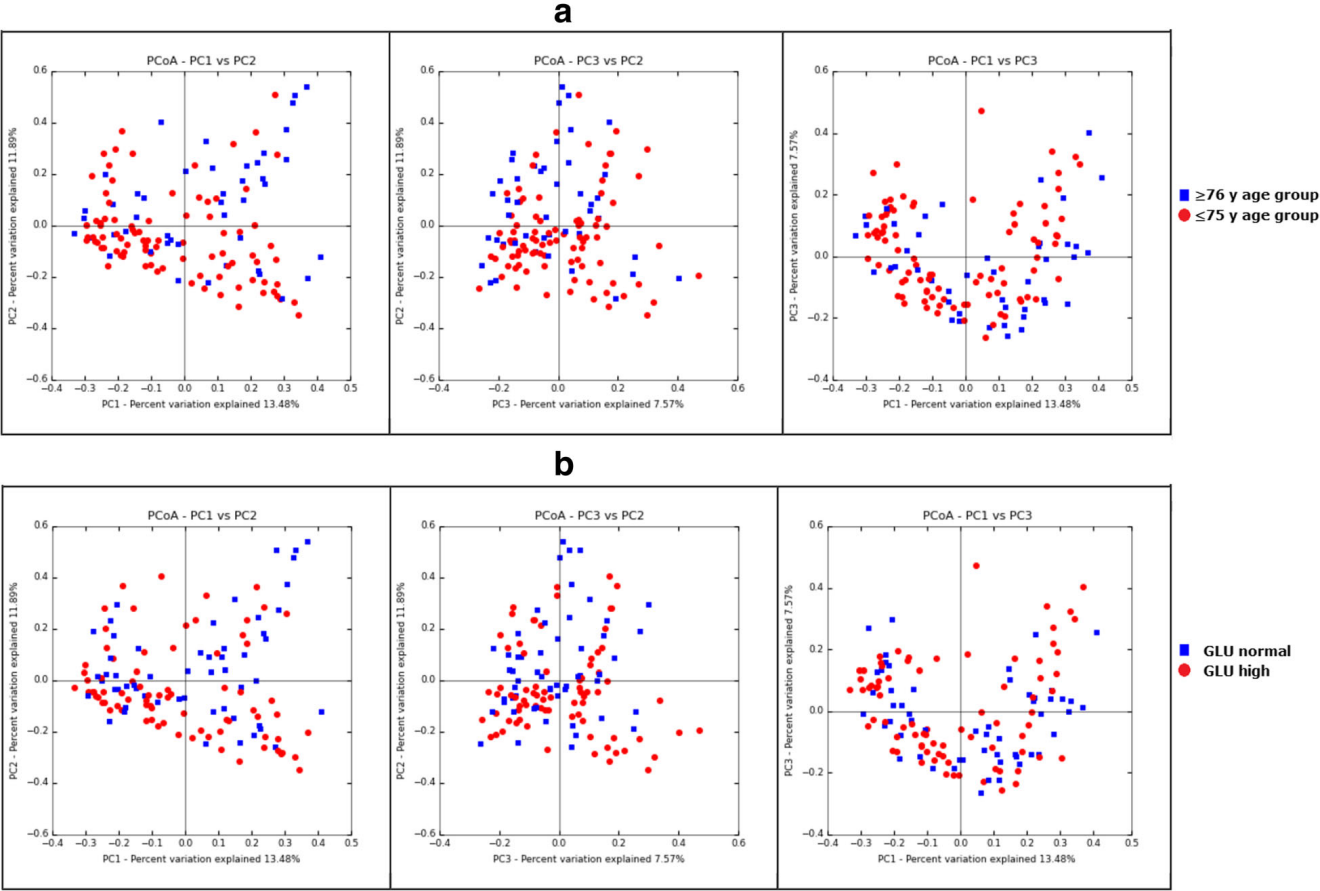
The 2D PCoA plots of the stool microbiota of people from two age group and different glucose levels based on Bray-Curtis distance matrix. (**a**) Comparison between the ≥ 76 y age group and ≤ 75 y age group. (**b**) Comparison between the GLU normal group and GLU high group

To understand the effects of subjects’ demographical, clinical, and behavioral characteristics on gut microbiota, we performed db-RDA analysis of each variable using Bray-Curtis matrix. The result shows that only factor of age and blood glucose level (GLU) have significant impacts on the Bray-Curtis distance matrix (Table 2). Further testing of the partial db-RDA analysis, which allows the influence of a matrix of conditioning variables to be partialed-out prior to analysis and thus enables evaluation of independent impacts of each individual factor on the gut microbiota structure shows that both GLU and age had a significant independent impact on the Bray-Curtis distance. Notably, the age factor has the most significant impact on the Bray-Curtis distance matrix, which explained 0.9% of total variances, independently. The 2D PCoA plots of the stool microbiota of people from two age group and different glucose levels based on Bray-Curtis distance matrix was shown in Fig. 1.

**Table 2:** Analysis of the association of demographic, biochemical and behavior characteristics variants and the microbiota based on db-RDA and Partial db-RDA on Bray-Curtis distance matrix

	db-RDA		Partial db-RDA	
	Adjusted R2	*p*	Adjusted R2	*p*
Gender	0.000786	0.286		
**Age**	**0.009047**	**0.003**	**0.00905**	**0.001**
Edu3	0.000428	0.362		
Exercise	0.001449	0.248		
Meat	−0.00074	0.583		
Fruit	−0.00051	0.534		
Vegetable	0.001639	0.214		
Alcohol	−0.0008	0.54		
Smoke	0.001909	0.249		
Sleeping	−0.00198	0.809		
Sleepless	0.001241	0.293		
Stress	−0.00141	0.668		
SBP	0.001487	0.181		
DBP	−0.00023	0.494		
BMI	−0.00147	0.834		
WHR	0.000624	0.281		
**GLU**	**0.004232**	**0.017**	**0.00423**	**0.01**
CHO	0.003007	0.057		
HDLC	0.002124	0.11		
LDLC	0.000757	0.272		
TG	−0.00045	0.561		

Boldface indicate P< 0.05

### Age supervised clustering of the microbiota

**Fig. 2:**
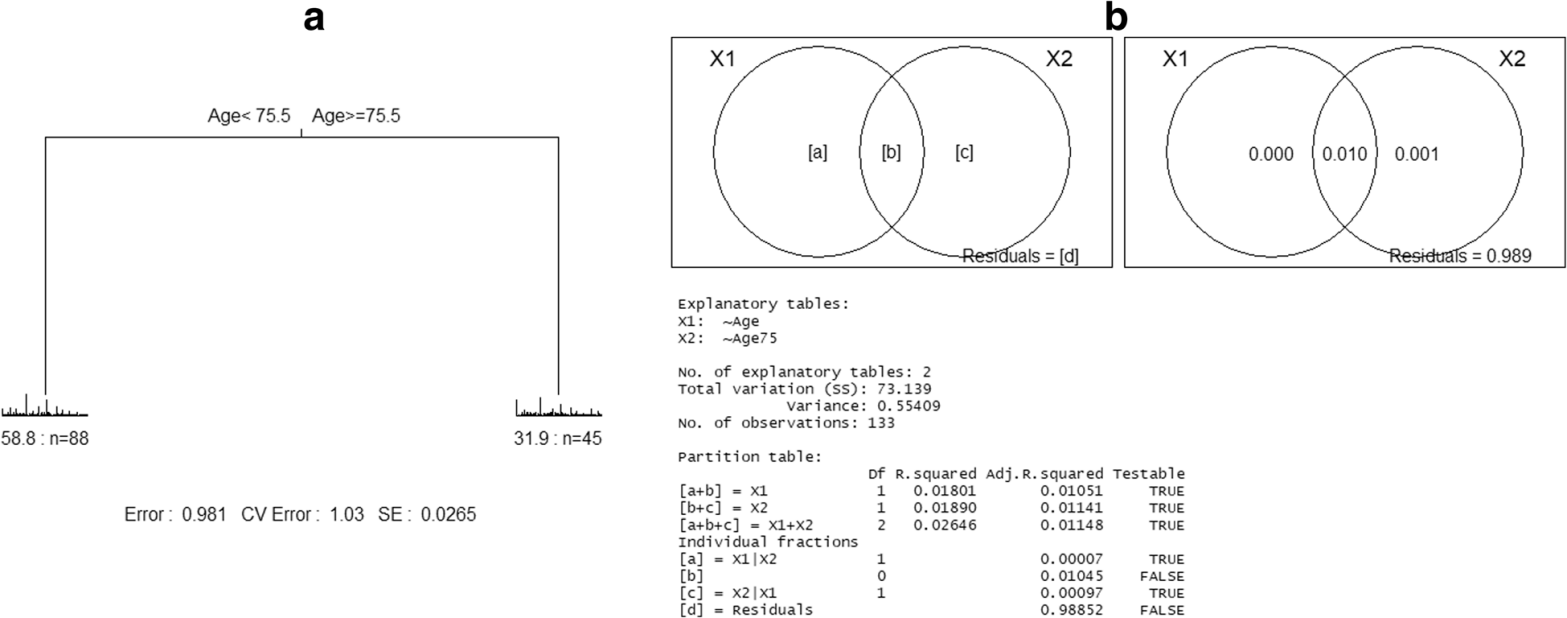
Clustering of the microbiota data supervised by the age based on MRT (multivariate regression trees) analysis and the result of variation partitioning analysis exploring explanatory power of numerical and categorial variant of age in relation to the microbiota data matrix. (**a**) The MRT analysis indicates that supervised by the age variant, the microbiota data could be stratified as 75 and younger group and 76 and older group. (**b**) The variation partitioning analysis indicates that the most part of the explanatory power of the numerical variant of age in relation to the microbiota data matrix could be explained by the two categorial variant of age (age of ≤75 or ≥ 76)

To investigate the impact of the age on the gut microbiota composition, we analyzed bacterial differentiated abundance using age-supervised multivariate regress trees (MRT). The results showed that when supervised by age, the microbiota could be stratified into two groups with 75.5 years as the cut-off age (Fig. 2).

Variation partitioning analysis was conducted to explore the explanatory power of a numerical age variant or a categorical age variant. The results indicated that using a categorical age variant with 75.5 years as the cut-off value could explain the majority of age-related gut microbiota changes.

### Comparison of diversity of microbial communities among groups with different glucose levels and age levels

**Fig. 3:**
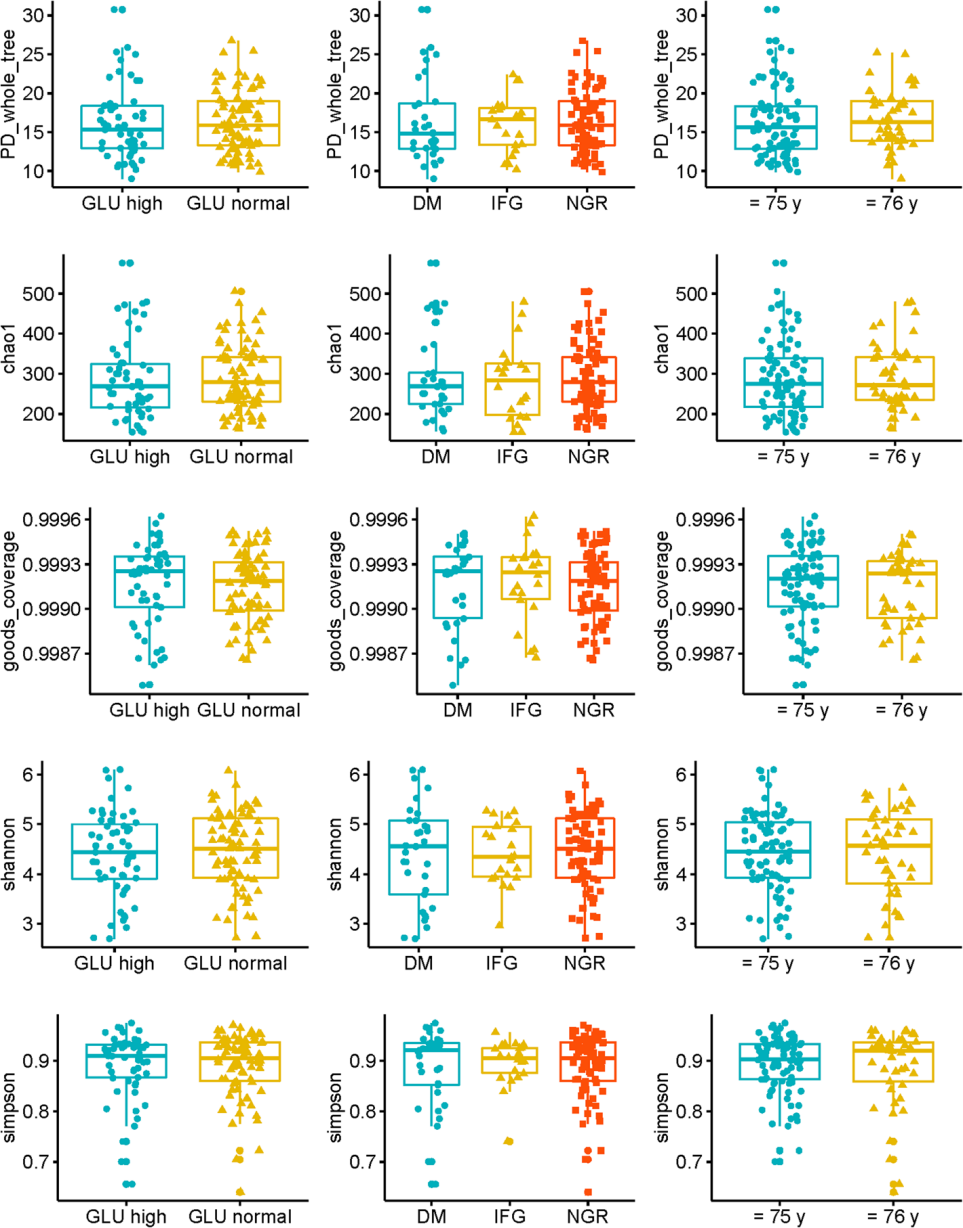
Comparison the alpha diversity between different groups of glucose levels and age levels

To compare the alpha diversity, the Chao1, Shannon, Simpson, PD whole tree, and Good’s coverage indexes were calculated after randomly subsampling the OTU table down to 60,265 reads per sample, the size of the smallest sample to obtain equal sequencing depth. No significant differences were found between different groups of glucose levels and age levels (age of ≤75 y or ≥ 76 y) (Table 3, Fig. 3).

**Table 3:** Comparison of the alpha diversity indexes across different group of samples

Overall comparison												
	Two Categorical GLU group		Three Categorical GLU group			Age group		Total (*n* = 133)				
	GLU normal (*n* = 78)	GLU high (*n* = 55)	NGR (*n* = 78)	IFG (*n* = 22)	DM (*n* = 33)	76 or older (*n* = 45)	75 or younger (*n* = 88)	min	median	max	mean	sd
PD_whole_tree	16.4 ± 4	16.2 ± 4.6	16.4 ± 4	16 ± 3.5	16.3 ± 5.3	16.6 ± 3.8	16.2 ± 4.5	9	15.8	30.7	16.3	4.2
Chao1	290.3 ± 82.7	286.2 ± 98.3	290.3 ± 82.7	278.6 ± 92	291.3 ± 103.4	291.4 ± 81.9	287.2 ± 93	154.5	272.2	576.5	288.6	89.1
Goods_coverage	1 ± 0	1 ± 0	1 ± 0	1 ± 0	1 ± 0	1 ± 0	1 ± 0	1	1	1	1	0
Shannon	4.5 ± 0.8	4.4 ± 0.9	4.5 ± 0.8	4.4 ± 0.6	4.4 ± 1	4.4 ± 0.9	4.5 ± 0.8	2.7	4.5	6.1	4.4	0.8
Simpson	0.9 ± 0.1	0.9 ± 0.1	0.9 ± 0.1	0.9 ± 0	0.9 ± 0.1	0.9 ± 0.1	0.9 ± 0.1	0.6	0.9	1	0.9	0.1
Comparison among 76 ages and older subgroup												
	Two Categorical GLU group		Three Categorical GLU group				Total (*n* = 45)					
	GLU normal (*n* = 26)	GLU abnormal (*n* = 19)	NGR (*n* = 26)		IFG (*n* = 6)	DM (*n* = 13)	min	median	max	mean	sd	
PD_whole_tree	17.2 ± 3.9	15.8 ± 3.7	17.2 ± 3.9		15.8 ± 3.7	15.8 ± 3.9	9.0	16.3	25.2	16.6	3.8	
Chao1	301.3 ± 81.9	278 ± 82.1	301.3 ± 81.9		262.7 ± 110.6	285 ± 69.7	162.3	272.1	479.5	291.4	81.9	
Goods_coverage	1 ± 0	1 ± 0	1 ± 0		1 ± 0	1 ± 0	1.0	1.0	1.0	1.0	0.0	
Shannon	4.6 ± 0.8	4.2 ± 0.9	4.6 ± 0.8		4.2 ± 0.8	4.2 ± 1	2.7	4.6	5.7	4.4	0.9	
Simpson	0.9 ± 0.1	0.9 ± 0.1	0.9 ± 0.1		0.9 ± 0.1	0.9 ± 0.1	0.6	0.9	1.0	0.9	0.1	
Comparison among 75 ages and younger subgroup												
	Two Categorical GLU group		Three Categorical GLU group				Total (*n* = 88)					
	GLU normal (*n* = 52)	GLU abnormal (*n* = 36)	NGR (*n* = 52)		IFG (*n* = 16)	DM (*n* = 20)	min	median	max	mean	sd	
PD_whole_tree	16 ± 4	16.4 ± 5.1	16 ± 4		16.1 ± 3.6	16.6 ± 6.1	9.9	15.6	30.7	16.2	4.5	
Chao1	284.8 ± 83.3	290.6 ± 106.7	284.8 ± 83.3		284.6 ± 87.3	295.4 ± 122	154.5	274.2	576.5	287.2	93.0	
Goods_coverage	1 ± 0	1 ± 0	1 ± 0		1 ± 0	1 ± 0	1.0	1.0	1.0	1.0	0.0	
Shannon	4.4 ± 0.7	4.5 ± 0.8	4.4 ± 0.7		4.5 ± 0.5	4.5 ± 1	2.7	4.5	6.1	4.4	0.8	
Simpson	0.9 ± 0.1	0.9 ± 0.1	0.9 ± 0.1		0.9 ± 0	0.9 ± 0.1	0.7	0.9	1.0	0.9	0.1	

In the analyses of beta diversity, consistent with the result of db-RDA analysis, Adonis tests also shows that both glucose level variance and age levels (age of ≤75 y or ≥ 76 y) were significantly associated with Bray-Curtis distance matrix. However, in the Adonis test after stratification into ≤75 y and ≥ 76 y age groups, the association between glucose levels and Bray-Curtis distance matrix for the≤75 y age group was no longer significant, while in the ≥76 y age group, the association was still significant with a significant higher R-square values (Table 4). These results suggested that the gut microbiota structure differed more significantly as glucose levels changed in the ≥76 y age group than in the ≤75 y age group.

**Table 4:** Adonis analysis on the association of microbiota and different glucose levels based on Bray-Curtis distance matrixes

	GLU normal VS GLU high		NGR VS IGF VS DM	
	*R* square value	*P* value	*R* square value	*P* value
**Overall data**	**0.01961**	**0.003**	**0.05439**	**0.002**
**Aged 76 and older group**	**0.09618**	**0.002**	**0.11801**	**0.002**
Aged 75 and younger group	0.01955	0.117	0.03661	0.091

Boldface indicate P< 0.05

**Fig. 4:**
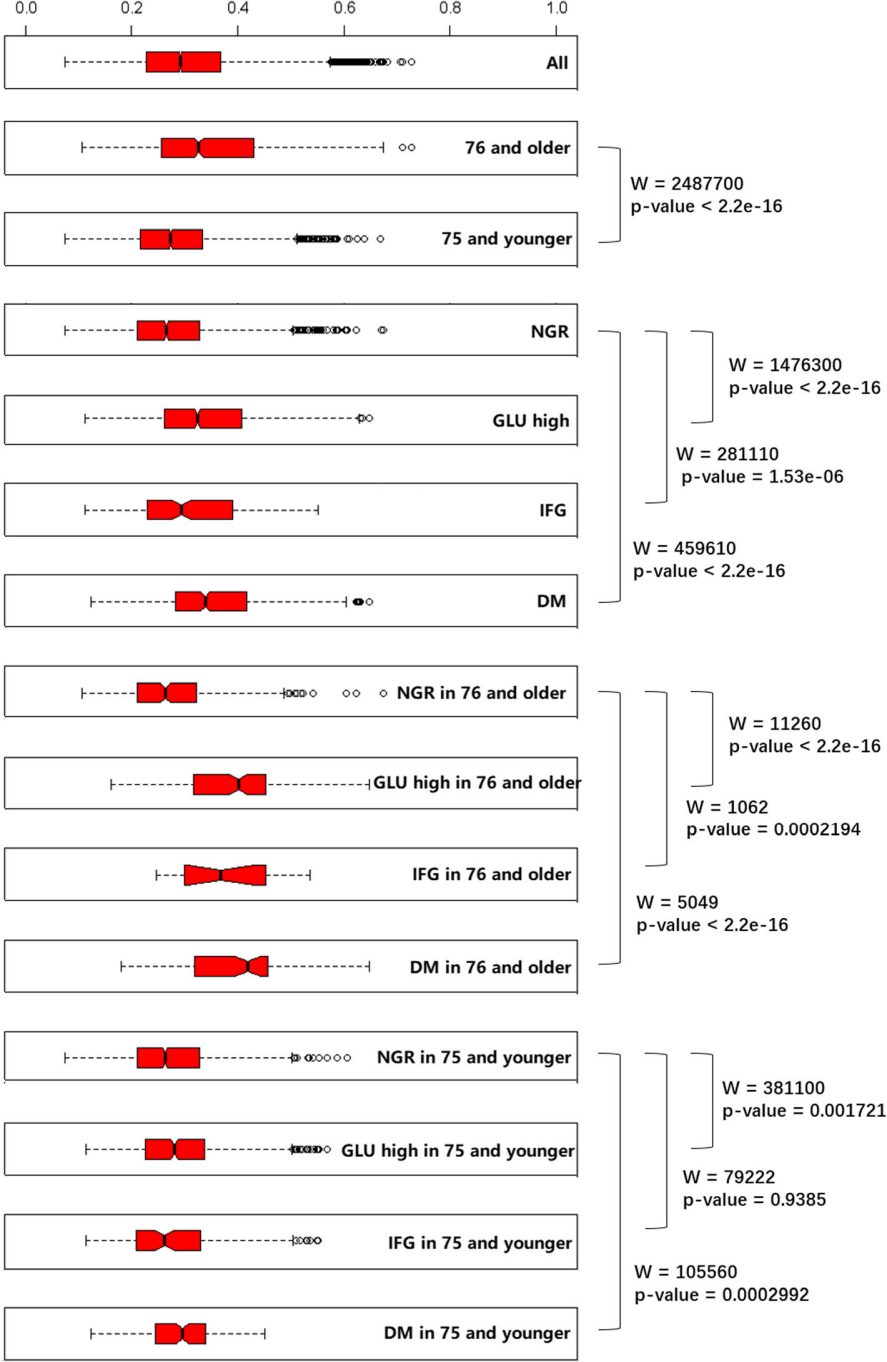
Comparison of inter-individual variation within group among samples from different groups of glucose levels stratified by age

We compared the microbiota Bray-Curtis distance matrix of groups with different ages and glucose levels using the Wilcoxon signed-rank test. The results showed that although inter-individual variation within groups was considerably high, that of the low glucose group was still significantly lower than that of the high glucose group, and that of the ≤75 y age group was significantly lower than that of the ≥76 y age group. After stratification into ≤75 y and ≥ 76 y age groups, the inter-individual variation of the high glucose group was significantly higher than that of the normal glucose group (Fig. 4).

### Differences in stool microbiota taxa between groups with different glucose levels

In Wilcoxon rank-sum test, a total of 9 taxa were found to have different abundances between the GLU normal group and GLU high group (*q* < 0.05 after correction for multiple testing by false discovery rate (FDR) control with the Benjamini–Hochberg procedure) (Table 5). Among them, the Actinobacteria at phylum level, Actinobacteria and Bacilli at class level, Bifidobacteriales, and Lactobacillales at order level, Bifidobacteriaceae and Lactobacillaceae at family level, and *Lactobacillus* and *Bifidobacterium* at genus level were more abundant in the GLU high group.

**Table 5:** Differences in stool microbiota taxon between different groups of glucose levels

Overall comparison												
Taxon	GLU normal (Mean ± SD)	GLU high (Mean ± SD)	Wilcoxon rank-sum test		Discriminatory power		GLU normal (Mean ± SD)	DM (Mean ± SD)	Wilcoxon rank-sum test		Discriminatory power	
			*p*-value	*q*-value	AUC value	sig.			*p*-value	*q*-value	AUC value	sig.
p__Actinobacteria	0.0253 ± 0.0363	0.0761 ± 0.0921	0.0003	0.018	0.6848	0.0004	0.0253 ± 0.0363	0.0846 ± 0.088	0	0.0028	0.7463	0.0003
p__Actinobacteria.c__Actinobacteria	0.0253 ± 0.0363	0.0761 ± 0.0921	0.0003	0.018	0.6848	0.0004	0.0253 ± 0.0363	0.0846 ± 0.088	0	0.0028	0.7463	0.0003
p__Actinobacteria.c__Actinobacteria.o__Bifidobacteriales	0.0161 ± 0.026	0.0631 ± 0.0815	0.0001	0.0099	0.6976	0.0003	0.0161 ± 0.026	0.0714 ± 0.0855	0	0.0028	0.7475	0.0004
p__Actinobacteria.c__Actinobacteria.o__Bifidobacteriales.f__Bifidobacteriaceae	0.0161 ± 0.026	0.0631 ± 0.0815	0.0001	0.0099	0.6976	0.0003	0.0161 ± 0.026	0.0714 ± 0.0855	0	0.0028	0.7475	0.0004
p__Actinobacteria.c__Actinobacteria.o__Bifidobacteriales.f__Bifidobacteriaceae.g__Bifidobacterium	0.0161 ± 0.026	0.062 ± 0.0803	0.0001	0.0099	0.6969	0.0003	0.0161 ± 0.026	0.0696 ± 0.084	0	0.0028	0.7457	0.0004
p__Firmicutes.c__Bacilli	0.0033 ± 0.0069	0.0357 ± 0.0942	0.0008	0.042	0.6714	0.0021	0.0033 ± 0.0069	0.0315 ± 0.0799	0.001	0.0476	0.6981	0.0028
p__Firmicutes.c__Bacilli.o__Lactobacillales	0.0032 ± 0.0069	0.0353 ± 0.0923	0.0009	0.0444	0.669	0.0021	0.0032 ± 0.0069	0.0315 ± 0.0799	0.001	0.0476	0.6985	0.0027
p__Firmicutes.c__Bacilli.o__Lactobacillales.f__Lactobacillaceae	0.0011 ± 0.0053	0.0271 ± 0.0812	0	0.0099	0.7064	0.0155	0.0011 ± 0.0053	0.0238 ± 0.0752	0	0.0028	0.7479	0.0268
p__Firmicutes.c__Bacilli.o__Lactobacillales.f__Lactobacillaceae.g__Lactobacillus	0.0011 ± 0.0053	0.0271 ± 0.0812	0	0.0099	0.7064	0.0155	0.0011 ± 0.0053	0.0238 ± 0.0752	0	0.0028	0.7479	0.0268
Comparison among 76 ages and older subgroup												
Taxon	GLU normal (Mean ± SD)	GLU high (Mean ± SD)	Wilcoxon rank-sum test		Discriminatory power		GLU normal (Mean ± SD)	DM (Mean ± SD)	Wilcoxon rank-sum test		Discriminatory power	
			*p*-value	*q*-value	AUC value	sig.			*p*-value	*q*-value	AUC value	sig.
p__Actinobacteria	0.0293 ± 0.0363	0.1322 ± 0.0917	0	0.0004	0.8725	0.0011	0.0293 ± 0.0363	0.1581 ± 0.0934	0	0.0002	0.926	0.0017
p__Actinobacteria.c__Actinobacteria	0.0293 ± 0.0363	0.1322 ± 0.0917	0	0.0004	0.8725	0.0011	0.0293 ± 0.0363	0.1581 ± 0.0934	0	0.0002	0.926	0.0017
p__Actinobacteria.c__Actinobacteria.o__Bifidobacteriales	0.0231 ± 0.0348	0.1169 ± 0.0907	0	0.0004	0.8725	0.0015	0.0231 ± 0.0348	0.1384 ± 0.0959	0	0.0002	0.9201	0.0023
p__Actinobacteria.c__Actinobacteria.o__Bifidobacteriales.f__Bifidobacteriaceae	0.0231 ± 0.0348	0.1169 ± 0.0907	0	0.0004	0.8725	0.0015	0.0231 ± 0.0348	0.1384 ± 0.0959	0	0.0002	0.9201	0.0023
p__Actinobacteria.c__Actinobacteria.o__Bifidobacteriales.f__Bifidobacteriaceae.g__Bifidobacterium	0.0231 ± 0.0348	0.1164 ± 0.09	0	0.0004	0.8745	0.0015	0.0231 ± 0.0348	0.1378 ± 0.0951	0	0.0002	0.9201	0.0022
p__Bacteroidetes.c__Bacteroidia.o__Bacteroidales.f__Bacteroidaceae	0.29 ± 0.1638	0.1333 ± 0.1286	0.0015	0.0464	0.7733	0.0052	0.29 ± 0.1638	0.1249 ± 0.1186	0.0028	0.0867	0.7899	0.0094
p__Bacteroidetes.c__Bacteroidia.o__Bacteroidales.f__Bacteroidaceae.g__Bacteroides	0.29 ± 0.1638	0.1333 ± 0.1286	0.0015	0.0464	0.7733	0.0052	0.29 ± 0.1638	0.1249 ± 0.1186	0.0028	0.0867	0.7899	0.0094
p__Firmicutes.c__Bacilli	0.0032 ± 0.0053	0.0822 ± 0.1438	0.0001	0.0042	0.834	0.0047	0.0032 ± 0.0053	0.068 ± 0.1172	0.0002	0.0091	0.8491	0.0048
p__Firmicutes.c__Bacilli.o__Lactobacillales	0.0032 ± 0.0053	0.0811 ± 0.1406	0.0001	0.0042	0.832	0.0047	0.0032 ± 0.0053	0.0679 ± 0.1171	0.0002	0.0091	0.8491	0.0048
p__Firmicutes.c__Bacilli.o__Lactobacillales.f__Lactobacillaceae	0.0012 ± 0.0037	0.0647 ± 0.1244	0	0.0001	0.9069	0.0259	0.0012 ± 0.0037	0.0574 ± 0.1116	0	0.0002	0.9201	0.0262
p__Firmicutes.c__Bacilli.o__Lactobacillales.f__Lactobacillaceae.g__Lactobacillus	0.0012 ± 0.0037	0.0647 ± 0.1244	0	0.0001	0.9069	0.0259	0.0012 ± 0.0037	0.0574 ± 0.1116	0	0.0002	0.9201	0.0262
p__Firmicutes.c__Clostridia.o__Clostridiales.f__Lachnospiraceae	0.233 ± 0.12	0.1087 ± 0.0799	0.0003	0.0153	0.8057	0.0031	0.233 ± 0.12	0.0925 ± 0.0574	0.0002	0.0091	0.855	0.0048
p__Firmicutes.c__Clostridia.o__Clostridiales.f__Lachnospiraceae.g__Lachnospiracea_incertae_sedis	0.0305 ± 0.026	0.0107 ± 0.0099	0.0014	0.0464	0.7753	0.0147	0.0305 ± 0.026	0.01 ± 0.0094	0.0028	0.0867	0.7899	0.0303

The discriminatory power of taxa was further assessed by calculating the AUC of a logistic regression model. As a result, the AUC values of all 9 taxa were above 0.65, and among them, the taxa *Lactobacillus* at genus level and Lactobacillaceae at family level were shown to have AUC values higher than 0.7, which represents reasonable discrimination power.

The HGR was further divided into IFG and DM subgroups, which were then compared with the NGR group. No taxa were found have different abundances between the NGR group and IFG group, after correction for multiple testing by FDR control with the Benjamini–Hochberg procedure. In the comparison of the NGR group and DM group, all the 9 taxa, which were more abundant in the GLU high group comparing to GLU normal group were also found have different abundances (*q* < 0.05 after correction for multiple testing by FDR control with the Benjamini–Hochberg procedure). The AUC values of the 9 taxa ranged from 0.69 to 0.75, and the AUC values of taxa Actinobacteria at phylum level, Actinobacteria at class level, Bifidobacteriales at order level, Bifidobacteriaceae and Lactobacillaceae at family level, and *Bifidobacterium* and *Lactobacillus* at genus level were higher than 0.7, which represents reasonable discrimination power.

### Comparison of stool microbiota taxa between groups with different glucose levels after age-stratification using 76 years as the cut-off age

We compared differentiated taxa in groups with different glucose levels after stratification with 75-years as the cut-off age using the Wilcoxon rank-sum test (Table 5). The results showed no differentiated taxa between groups with different glucose levels in the ≤75 y age group. However, in the ≥76 y age group, 13 taxa showed differentiated abundances between the high GLU group and normal GLU group (*q* < 0.05 after correction for multiple testing by FDR control with the Benjamini–Hochberg procedure). The Bacteroidaceae and Lachnospiraceae at family level, and Lachnospiraceae *incertae sedis* and *Bacteroides* at genus level were more abundant in the GLU normal group, whereas the Actinobacteria at phylum level, Actinobacteria and Bacilli at class level, Bifidobacteriales and Lactobacillales at order level, Lactobacillaceae and Bifidobacteriaceae at family level, and *Lactobacillus* and *Bifidobacterium* at genus level were more abundant in the GLU high group. All had AUC values greater than 0.80. Among the 13 taxa, Lactobacillaceae and *Lactobacillus* had AUC values greater than 0.90, which suggested these two taxa had satisfactory discrimination power. Among those 13 taxa, 10 taxa also showed differentiated abundances between NGR and DM groups with AUC values greater than 0.85. no taxa showed differentiated abundances between the NGR and IFG group.

## Discussion

In this study, we investigated the association between gut microbiota and variables including plasma glucose levels and demographic, behavioral, and biochemical characteristics in a population with chronic disease. The results indicated that plasma glucose level and age contributed significantly to a differentiated gut microbiota structure. We also identified several taxa whose abundance differed significantly in subgroups with different glucose levels. After age stratification, we found that the plasma glucose level affected the gut microbiota structure more significantly in the ≥76 y age group than in the ≤75 y age group. Moreover, the taxon abundance changed significantly with glucose level in different age groups.

With the widespread application of next-generation sequencing technology, more and more studies have demonstrated a close relationship between gut microbiota and human health and disease. Multiple host demographic factors and behavioral factors play important and confounding roles in the relationship between gut microbiota and physio-pathological indicators.

Our study analyzed the association between gut microbiota structure and glucose level and multiple demographic, behavioral, and biochemical factors using db-RDA. The results demonstrated that age and glucose level were significantly associated with gut microbiota structure.

Several studies have investigated the relationship between age or glucose level and microbiota composition, and several hypotheses have been proposed. During the aging process, the physiology of the intestinal tract is affected, dietary habits and lifestyles change, and immunosenescence occurs, all of which contribute to age-related imbalance of the intestinal microbial community. Reported age-related changes in the intestinal microbiota include dysbiosis, loss of microbial diversity, increased vulnerability to environmental perturbations, loss of probiotics, shifts in the dominant species within several bacterial groups, increase in the total number of facultative anaerobes, and reduced SCFA production rates. These modifications of the intestinal microbiota may contribute to risk for several diseases like inflammatory bowel conditions, metabolic diseases, as well as musculoskeletal conditions [24]. Previous studies have suggested that abnormal blood glucose levels might cause gut microbiota changes, such as dysbacteriosis, compositional changes of microbiota, and changes in metabolites. For example, as the blood glucose level changes, the concentration of short-chain fatty acids (SCFAs), which have significant immune system effects in the intestinal mucosa, decreases, while the concentration of LPS from gram-negative bacteria increases. As a result, the pro-inflammatory signal transduction pathway is activated and consequently causes chronic low-grade inflammatory status, reduced insulin sensitivity, and a series of changes that eventually lead to the occurrence of T2D [25, 26]. All of these studies indicate that dysbacteriosis is closed associated with aging and abnormal blood glucose levels [25, 26]. However, an objective and comprehensive definition of intestinal flora imbalances is still lacking.

Some studies have attempted to identify the alpha-diversity related characteristics in the gut microbiota of aged populations. Biage et al. observed that the microbial composition and diversity of the gut ecosystem of young adults differs significantly from that of Italian centenarians [27]. However, Bian et al. and Kong et al. reported that the microbiota of healthy aged adults differs little from that of healthy young adults in the Chinese population [28, 29]. Similarly, it is debatable whether alpha-diversity related microbiota characteristics are associated with abnormal blood glucose levels, with some studies reporting associations between lower microbiota diversity and T2D or insulin resistance [16, 30], while others do not support such associations [14, 17, 20]. In this study, we compared five alpha-diversity indices between different age groups with various blood glucose levels and did not find significant differences. Discrepancies in the association between microbiota alpha-diversity and age or blood glucose levels could be explained by ethnic or demographic difference between studies. For example, Wang et al. investigated and compared the composition and richness of the gut microbiota of healthy individuals and diabetes patients from two ethnic groups, Uyghurs and Kazaks. Significant differences in microbial richness and a higher number of OTUs were found between the Kazak healthy and diabetic groups, while no major differences in intestinal microbiota were found between the Uyghur healthy and diabetic groups [13]. Another possible explanation might be that alpha-diversity is affected by multiple confounding factors. When the factors are not well controlled, the results might be biased.

Inter-individual variation within groups is another parameter that can reflect the stability of gut microbiota. Individuals with healthy and hemostatic gut microbiota share higher similarity regarding the composition and richness of microbes, while those with imbalanced microbiota show different changes and tend to have higher inter-individual variations. This is similar to the so-called Anna Karenina principle, derived from Leo Tolstoy’s dictum that “all happy families look alike; each unhappy family is unhappy in its own way”, and has been used for successful modeling in many different fields such as business, psychology, economics, biology, and recently in microbiota [31]. In this study, we compared the inter-group differences of the distances matrix under different age and plasma glucose conditions using the Wilcoxon signed-rank test. The results showed considerable inter-individual variation within groups. However, the inter-individual variation of the low blood glucose group was significantly lower than that of the high blood glucose group, and the inter-individual variation of the ≤75 y age group was significantly lower than that of the ≥76 y age group. These results are consistent with Bian’s report [28] in which the 94-year-old group had a larger beta diversity than did younger groups, and with Qin’s report [14] in which T2D was found to be a significant factor in the variation in examined gut microbial samples. These studies all support the hypothesis that microbiota homeostatic imbalance is age and blood sugar level-related. The elderly or individuals with abnormal blood sugar levels tend to have higher inter-individual variation in gut microbes or the variation tends to be greater.

Further Wilcoxon signed-rank tests in the age-stratified groups showed that although the inter-individual variation of the high-blood glucose group was significantly higher than that of NGP in both ≤75 y and ≥ 76 y age groups, compared with the ≤75 y age group, the differences of the inter-individual variation were more significant in the ≥76 y age group. This result is consistent with the Adonis analysis of the age-stratified groups and indicated that the association between blood glucose level and gut microbiota stability differs in different age groups. Therefore, it is necessary to consider the effects of age when investigating the relationship between blood glucose and gut microbiota.

In this study, we analyzed the taxonomic differences in gut microbiota in groups with different blood glucose levels. We found that *Lactobacillus* species and their related taxa had higher richness in the high blood glucose group (> 6.0) as compared with the normal blood glucose group (≤6.0). Similarly, *Lactobacillus* species and their related taxa had higher richness in the DM group than in the NGT group. This is consistent with previous studies on diabetic subjects in different populations of the world indicating a significantly higher abundance of *Lactobacillus* species in fecal samples of high blood glucose groups [16, 19, 20]. In children with insulin-dependent diabetes mellitus (IDDM), high salivary glucose levels lead to increased salivary lactobacilli counts [32]. Therefore, increased abundance of *Lactobacillus* in the gastrointestinal tract could be the result of increased intestinal glucose levels [16]. In fact, our results also demonstrated that the abundance of *Lactobacillus* tends to increase as the blood glucose level increases in NGT, IFG, and DM groups.

The richness of *Bifidobacterium* species and their related taxa was also found significantly associated with elevated blood glucose levels. This result is contrary to those of Wu et al. [18] and Sedighi et al. [19]. They found lower concentrations of *Bifidobacterium* in T2DM patients than in normal controls. However, our result is consistent with that of Sepp et al., in which the counts and proportions of *Bifidobacterium* were associated with higher glucose levels [15]. Therefore, the association between the richness of *Bifidobacterium* species and blood glucose levels is controversial and needs further investigation.

The controversy over the association between gut microflora composition and blood glucose levels could be explained by differences in the participating populations, the microbial detection methods, the statistical methods, etc. In addition, multiple confounding factors could cause contradictory results. In our study, we can conclude that age is an important factor affecting the association between blood glucose levels and gut microbial composition.

It is generally accepted that an age of 60 or 65 years is defined as elderly or old. However, the definition of aged gut microbiota is still debatable. Current investigations of the association between age and gut microbiota are mainly based on different age groups, but the results vary significantly [27, 28, 33, 34]. When conducting age-related microbiota studies, using study-specific age standard may improve the reliability of the results. Therefore, we used a supervised clustering method and found the microbiota could be strictly stratified into two groups with 75.5 years as the cut-off age. Further variation partitioning analysis shows that the categorical age variant with 75.5 years as the cut-off value could explain the majority of age-related gut microbiota changes, therefore the age of 75.5 years might be the best cut-off to stratify the population of this study. The finding of the potential switch in the microbiota structure at the age of 76 years in this study population is very interesting and indicative, however more researches must be conducted to evaluate whether the cut off age of 75.5 years is also applicable to other populations.

After stratification by age at 75 years, the differentiated taxa were analyzed and compared in groups with different blood glucose levels. The result showed that the blood glucose-related taxa differed significantly between the ≤75 y and ≥ 76 y age groups. In the ≤75 y age group, the differences of blood glucose-related taxa showed a similar trend as in the ≥76 y age group. However, after correcting by q value, the difference was not significant (data not shown). In the ≥76 y age group, the differences of these taxa were much more significant. For example, *Lactobacillus* genus and 3 related taxa, as well as *Bifidobacterium* genus and 5 related taxa, were able to differentiate high glucose and normal glucose, as well as DM and NGT, with an AUC value greater than 0.8.

In addition, in the ≥76 y age group, 2 taxa, the Lachnospiraceae family and the *Lachnospiracea incertae sedis* genus showed significantly higher abundances in the normal blood glucose group. Of them, the Lachnospiraceae family also displayed significant associations with blood glucose level when we compared the DM and NGR groups. An association between the Lachnospiraceae family and blood glucose level has been reported, but the results are inconsistent. According to Bhute et al. [16], Lachnospiraceae were significantly more abundant in NGTs subjects than in the DM group, while Qin et al. reported that an metagenomic linkage group (MLG) assigned to the Lachnospiraceae family was significantly associated with T2D [14]. In our study, the association between Lachnospiraceae and its related taxa and blood glucose levels were not significant in the ≤75 y age group (*p* > 0.05), but very significant in ≥76 y age DM group with all the AUC values greater than 0.85 (*p* < 0.01, *q* > 0.05). These results suggested that the associations between these taxa and blood glucose levels might change with different age levels.

There are some limitations of this study. First, due to the cross-sectional experimental design, we were not able to determine the causal connection between aging, abnormal glucose, and changes in gut microbiota. Second, we used 16 sRNA gene sequencing to analyze the gut microbiome. This method could introduce bias during several processes, including the selection of the gene amplification area, the gene amplification procedure, the selection of gene sequence database, and OTU clustering. In addition, this study included many elderly participants. Older people generally have a higher burden of multimorbidity and polypharmacy than younger ones, and both these elements could be associated with a different fecal microbiota composition. This could also introduce some bias in the results of this study. However, compared with other studies that have investigated the relationship between blood glucose level and gut microbiota, the strengths of our work includes, we enrolled participants with a wider age range, all the participants were from the same community and their behavioral and physiological parameters were collected.

## Conclusions

In this study, we found that both blood glucose level and age have significant impacts on the composition of gut microbiota. The association between glucose level and the composition and activity of gut microbiota was affected profoundly by age and displayed distinct characteristics at different age groups. Our findings suggest that it is necessary to take age into account when investigating the association between glucose and gut microbiota. We also identified multiple taxa that were highly associated with high glucose levels in the ≥76 y age group, but not in the ≤75 y age group. More research is required to determine the underlying biological mechanisms.

## Methods

### Sample collection and processing

With approval from the Ethics Committee of Minzu University of China (MUC), the subjects of the present study were enrolled from populations that participated in community health examinations in Beijing in August 2015. The inclusion criteria included 1) Subject is a male or female aged over 40. 2) Subject did not take any medicine in recent 2 weeks. 3) subject have no previous chronic gastrointestinal disease. The exclusive criteria included usage of any medicine, probiotics, or prebiotics within 2 weeks, diagnosis of psychiatric disorders, intestinal diseases, and neoplasia. A total of 133 subjects were enrolled in the study after obtaining both written and verbal consent from the subjects.

Height, weight, waist-to-hip ratio, and blood pressure were measured for all subjects, and risk factor information of lifestyle profile (such as exercise, diet, smoking, alcohol, sleeping, and stress) were collected by a questionnaire during the waiting time of the physical examination. Blood samples before breakfast were collected to measure blood lipids and glucose. Fecal samples were collected with sterile cups and were frozen at − 20 °C immediately, then transferred to the laboratory within 24 h and stored at − 80 °C before DNA extraction.

Blood samples before breakfast were tested for glucose, serum total cholesterol (CHO), low-density lipoprotein cholesterol (LDL-C), high-density lipoprotein cholesterol (HDL-C), and triglycerides (TG) using an automatic biochemical analyzer.

Extraction of total DNA from stool samples was conducted according to the manual of the PowerSoil® DNA Isolation Kit. The purity of DNA was evaluated using the A_260/280_ ratio. Samples with A_260/280_ ratios between 1.8 and 2.2 were used for further experiments.

### Microbiota sequencing

The V3-V4 region of the bacterial 16S rRNA gene was amplified with the common primer pair (forward primer, 5′- ACTCCTACGGGAGGCAGCA-3′; reverse primer, 5′- GGACTACHVGGGTWTCTAAT-3′) combined with adapter sequences and barcode sequences. PCR amplification was performed in a total volume of 50 μl, which contained 10 μl buffer, 0.2 μl Q5 High-Fidelity DNA Polymerase, 10 μl High GC Enhancer, 1 μl dNTP, 10 μM of each primer, and 60 ng genomic DNA. Thermal cycling conditions were as follows: an initial denaturation at 95 °C for 5 min, followed by 15 cycles at 95 °C for 1 min, 50 °C for 1 min, and 72 °C for 1 min, with a final extension at 72 °C for 7 min. The PCR products from the first step PCR were purified through VAHTSTM DNA Clean Beads. A second round of PCR was then performed in a 40-μl reaction which contained 20 μl 2 × Phusion HF MM, 8 μl ddH_2_O, 10 μM of each primer, and 10 μl PCR products from the first step. Thermal cycling conditions were as follows: an initial denaturation at 98 °C for 30 s, followed by 10 cycles at 98 °C for 10 s, 65 °C for 30 s min, and 72 °C for 30 s, with a final extension at 72 °C for 5 min. Finally, all PCR products were quantified by the Quant-iT™ dsDNA HS Reagent and pooled together. High-throughput sequencing analysis of bacterial rRNA genes was performed on the purified, pooled sample using the Illumina Hiseq 2500 platform (2 × 250 paired ends) at Biomarker Technologies Corporation, Beijing, China.

### Bioinformatics analysis

The raw reads were demultiplexed and then trimmed, merged, and filtered by Usearch9.0.2132_i86linux32 following the UPARSE pipeline. All reads were trimmed to the position of the first base with quality score ≤ 2, and sequences shorter than 64 after trimming were discarded. Paired reads with a number of expected error > 1.00 were further filtered out during the filtering step. Sequences were dereplicated and clustered with a threshold of 97% similarity for picking operational taxonomic units (OTUs) representative after Chimera checking. After that, all sequences were mapped back to the representative sequences resulting in an OTU table for all samples. The RDP Classifier was used to assign 16S rRNA gene sequences to a taxonomical hierarchy with a confidence threshold of 80%. OTU representative sequences were aligned and further filtered to create a phylogenetic tree using the QIIME pipeline. The OTU table was randomly subsampled down to the size of the smallest sample to obtain equal sequencing depth. Finally, a total of 60,265 reads per sample were used for further analysis.

### Statistical analysis

Categorical variables are presented as frequencies and percentages. Chi-squared tests and Fisher’s exact test were used to assess statistical associations between variables. Numerical variables are expressed as mean ± standard deviation (SD). ANOVA (one-way analysis of variance) was used to compare the differences between groups.

The Chao1, Shannon, Simpson, PD whole tree, and Good’s coverage indexes were used for richness and diversity estimations of the gut microbiota.

The db-RDA and Adonis tests were performed on the Bray-Curtis distance matrix to investigate the differences of beta diversity between different characteristics variables of the participants.

Multivariate regression tree methodology was used for cluster analysis of bacterial abundance where clusters were age stratified. This analysis enabled us to determine the age limit with the highest explanatory power.

The Wilcoxon rank-sum test were performed on abundance data to explore the taxa significantly different among groups. The discriminatory power of a taxon was further assessed by calculating the area under receiver-operating characteristic curve (AUC) of a logistic regression model.

## Abbreviations

ANOVA: One-way analysis of variance
AUC: Area under receiver-operating characteristic curve
CHO: Serum total cholesterol
DM: Diabetic group
FDR: False discovery rate
GLU: Blood glucose level
HDL-C: High-density lipoprotein cholesterol
HGR: High blood glucose group
IBD: Inflammatory bowel disease
IDDM: Insulin-dependent diabetes mellitus
IFG: Impaired fasting glucose group
LDL-C: Low-density lipoprotein cholesterol
LPS: Lipopolysaccharides
MLG: Metagenomic linkage group
MRT: Multivariate regress trees
MUC: Minzu University of China
NGR: Normal blood glucose group
OTUs: Operational taxonomic units
SCFAs: Short-chain fatty acids
SD: Standard deviation
T2D: Type 2 diabetes
TG: Triglycerides
TID: Type I diabetes
